# Efficient co-isolation of microvascular endothelial cells and satellite cell-derived myoblasts from human skeletal muscle

**DOI:** 10.3389/fbioe.2022.964705

**Published:** 2022-09-21

**Authors:** Rebecca Wüst, Lisanne Terrie, Thomas Müntefering, Tobias Ruck, Lieven Thorrez

**Affiliations:** ^1^ Tissue Engineering Lab, Dep. Development and Regeneration, KU Leuven Kulak, Kortrijk, Belgium; ^2^ Department of Neurology, Medical Faculty, Heinrich-Heine-University, Düsseldorf, Germany

**Keywords:** cell isolation, microvascular endothelial cells, myoblasts, co-isolation, human, skeletal muscle

## Abstract

Vascularization of tissue-engineered constructs remains a key challenge in the field of skeletal muscle tissue engineering. One strategy for vascularizing organoids is *in vitro* pre-vascularization, relying on *de novo* assembly of undifferentiated endothelial cells into capillaries, a process termed vasculogenesis. In most endothelial cell research to date, human umbilical vein endothelial cells have been used primarily because of their availability. Nevertheless, this endothelial cell type is naturally not occurring in skeletal muscle tissue. Since endothelial cells display a tissue-specific phenotype, it is of interest to use muscle-specific microvascular endothelial cells to study pre-vascularization in skeletal muscle tissue engineering research. Thus far, tissue biopsies had to be processed in two separate protocols to obtain cells from the myogenic and the endothelial compartment. Here, we describe a novel, detailed protocol for the co-isolation of human skeletal muscle microvascular endothelial cells and satellite cell-derived myoblasts. It incorporates an automated mechanical and enzymatic tissue dissociation followed by magnetically activated cell sorting based on a combination of endothelial and skeletal muscle cell markers. Qualitative, quantitative, and functional characterization of the obtained cells is described and demonstrated by representative results. The simultaneous isolation of both cell types from the same donor is advantageous in terms of time efficiency. In addition, it may be the only possible method to isolate both cell types as the amount of tissue biopsy is often limited. The isolation of the two cell types is crucial for further studies to elucidate cell crosstalk in health and disease. Furthermore, the use of muscle-specific microvascular endothelial cells allows a shift towards engineering more physiologically relevant functional tissue, with downstream applications including drug screening and regenerative medicine.

## 1 Introduction

In native skeletal muscle, vascular supply is provided by a highly organized extensive capillary network throughout the fibers to meet the high metabolic demands. The fundamental role of the microvascular network is to deliver oxygen and nutrients throughout the tissue. To vascularize engineered muscle, two core vascularization strategies are being explored. The first approach involves *in vivo* angiogenesis, which exploits the ingrowth of host vessels coming from sprouting endothelial cells (ECs) from pre-existing vessels. However, the rate of spontaneous ingrowth through angiogenesis is slow (approximately 5 µm/h) (Orr et al., 2003), which may limit the thickness of the tissue that can be transplanted. Since passive diffusion of metabolic products and gases is also limited, necrosis may occur before sprouts from the host blood vessels can reach the inner core. To overcome this, another strategy focuses on *in vitro* pre-vascularization through *de novo* blood vessel formation. This so-called vasculogenesis strategy uses ECs that coat the inner wall of vascular networks (Gholobova et al., 2020). The majority of research carried out to date, involving endothelial cells, uses human umbilical vein endothelial cells (HUVECs) (Wang et al., 2019). HUVECs are known to be robust and can be obtained from discarded umbilical cord tissue which is abundant. Also, their commercial availability is widespread and researchers can rely on years of accumulated knowledge (Hauseret al., 2017). HUVECs have the capacity to assemble into a capillary network within a 3-dimensional (3D) hydrogel, which was first described by Schechner et al. (2000), and afterwards extensively studied by others in the tissue engineering field. Indeed, also in muscle tissue engineering research, we have used HUVECs to study *in vitro* pre-vascularization thus far (Gholobova et al., 2015; Gholobova et al., 2019).

However, ECs are not only creating a passive conduit for blood delivery. Increasing insights in crosstalk between tissue-specific cell types (Rafii et al., 2016) and the growing evidence for the endothelium as a regulator of regenerative processes in an organ/tissue-specific manner (Rafii et al., 2016) promote the use of tissue-specific cells. These complex tasks are regulated by capillary EC-derived paracrine factors, which create an instructive organ-specific niche for repopulating stem and progenitor cells (Verma et al., 2018). In fact, satellite cells that express Pax7 and Myf5 were found to be positioned near ECs (Chiristov et al., 2007). In addition, the same study established that ECs specifically enhance satellite cell growth, while differentiating myogenic cells were found to be proangiogenic.

Thus, skeletal muscle microvascular endothelial cells (SkMVECs), which are naturally occurring in skeletal muscle, are of high interest for engineering pre-vascularized skeletal muscle. To date, only a few papers have been published on the isolation of SkMVECs. Chen et al. (1995) described a method for isolating primary rat SkMVECs avoiding enzymatic and mechanical isolation by outgrowth of the cells from tissue pieces within 60 h. Since fibroblasts and other cells are described to grow out of the muscle pieces only after 72 h, this would result in a pure population of SkMVECs. However, no clear numbers in terms of cell purity nor cell yield were given. Another protocol, focusing on murine skeletal muscle tissue, described an approach using fluorescence-activated cell sorting (FACS) to isolate Sca1^+^, CD31^+^, CD34^dim^ and CD45^−^ cells (Ieronimakis et al., 2008). In this protocol, the capacity of the isolated cells to take up acetylated low-density lipoprotein, to produce nitric oxide, and to form vascular tubes was described even after several passages in culture. However, in our specific case, magnetic-activated cell sorting (MACS) offered advantages compared to FACS such as a lower device cost, higher throughput and faster processing time (Tomlinson et al., 2013; Sutermaster and Darling 2019; Pan and Wan 2020). The most recent work on the isolation of primary murine SkMVECs was published by Müntefering et al. (2019). There, a protocol was established yielding CD31-positive murine SkMVECs with a purity of up to 95% using MACS (Müntefering et al., 2019). Taken together, only a few protocols have been published describing the isolation of primary SkMVECs and none of those has been applied to human tissue. Moreover, for co-culturing approaches in skeletal muscle tissue engineering, a protocol on the combined isolation of both primary myoblasts and primary SkMVECs from the same biopsy is not existing. Therefore, originally based on the protocol of Müntefering et al. (2019), we developed a protocol for the co-isolation of SkMVECs and satellite cell-derived myoblasts from the same human skeletal muscle tissue biopsy.

## 2 Materials and methods

**FIGURE 1:**
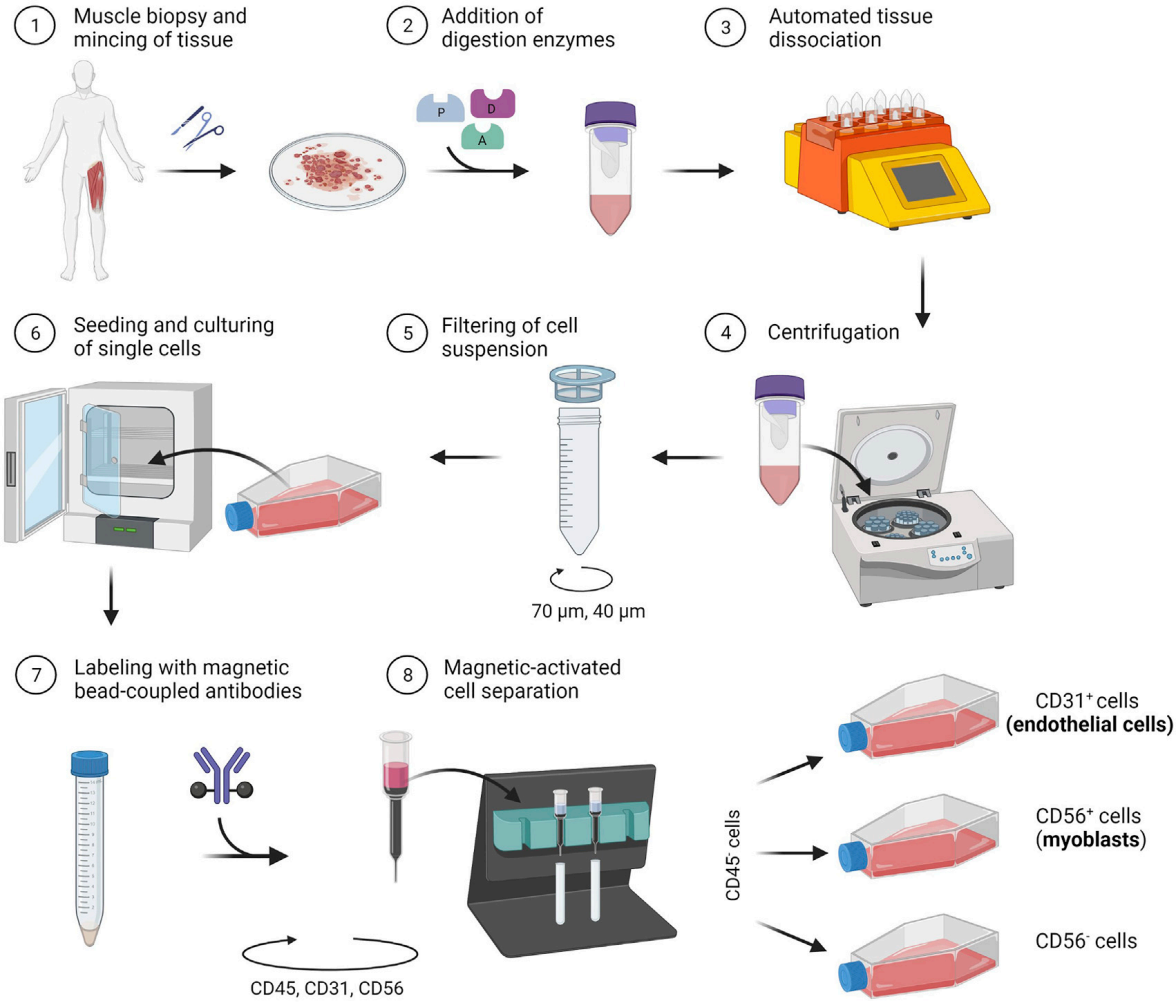
Schematic representation of the developed protocol for the co-isolation of microvascular endothelial cells (CD31^+^) and satellite cell-derived myoblasts (CD56^+^) from human skeletal muscle tissue.

**FIGURE 2:**
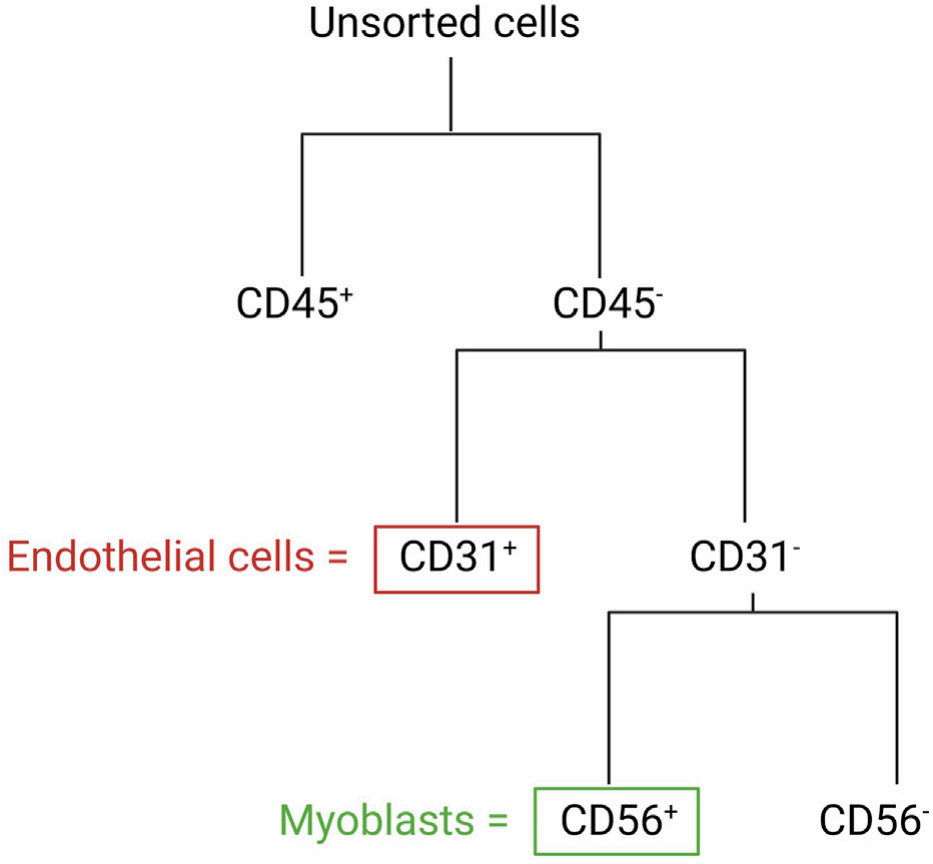
Schematic overview of the MAC sorting strategy to obtain endothelial cells (marker: CD31) and myoblasts (marker: CD56). Both cell types are isolated from the CD45-negative cell population (depleted cells of hematopoietic origin).

In the following section, we describe a detailed protocol for the co-isolation and characterization of SkMVECs and satellite cell-derived myoblasts from human skeletal muscle tissue. Fresh human muscle biopsies were obtained from the Human Body Donation Program of KU Leuven, at campus KULAK, Belgium (ethical approval number: NH019-2020-04-02). A schematic overview of the protocol is represented in Figure 1 (created with BioRender.com). The magnetic-activated cell sorting strategy for the co-isolation of endothelial cells and myoblasts is displayed in more detail in Figure 2. Lastly, a detailed list of used materials and reagents can be found in Table 1.

**TABLE 1:** Materials and reagents.

**Materials**	
Cell strainers, 40-µm	(VWR Corning, catalog number: 734-2760)
Cell strainers, 70-µm	(VWR Corning, catalog number: 734-2761)
GentleMACS™ C Tubes	(Miltenyi Biotec, catalog number: 130-093-237)
Large magnetic columns	(Miltenyi Biotec, catalog number: 130-042-401)
MACS^®^ MultiStand	(Miltenyi Biotec, catalog number: 130-042-303)
Medium magnetic columns	(Miltenyi Biotec, catalog number: 130-042-201)
MidiMACS™ Separator	(Miltenyi Biotec, catalog number: 130-042-302)
MiniMACS™ Separator	(Miltenyi Biotec, catalog number: 130-042-102)
**Reagents**	
Aprotinin	(Carl Roth, catalog number: A1624)
Bovine serum albumin (BSA)	(Sigma-Aldrich, catalog number: A2153)
CD31 MicroBeads, human	(Miltenyi Biotec, catalog number: 130-091-935)
CD45 MicroBeads, human	(Miltenyi Biotec, catalog number: 130-045-801)
CD56 MicroBeads, human	(Miltenyi Biotec, catalog number: 130-055-401)
Calcein AM	(Hello Bio, catalog number: HB0720)
Collagenase II	(Worthington Biochemical Corp., catalog number: LS004176)
DAPI	(Thermo Fisher, catalog number: D1306)
Dispase II	(Roche Diagnostics, catalog number: 4942078001)
Dulbecco’s Modified Eagle Medium (DMEM)	(Biowest, catalog number: L0103-500mL)
EDTA	(Sigma-Aldrich, catalog number: 2854)
EGF human	(Peprotech, catalog number: AF-100-15)
EGM-MV^TM^	(Lonza, catalog number: CC-4147)
Fetal Bovine Serum (FBS)	(Biowest, catalog number: SS 1810-500)
Fibrinogen	(Merck, catalog number: 341576)
Gentamicin	(Gibco, catalog number: 15750037)
Growth factor reduced Matrigel	(BD Biosciences, catalog number: 354230)
Insulin	(Sigma-Aldrich, catalog number: I9278-5ML)
KHCO_3_	(Sigma-Aldrich, catalog number: 1.04854)
Methylcellulose	(Carl Roth, catalog number: 8421.1)
NH_4_Cl	(Sigma-Aldrich, catalog number: 12125-02-9)
Phosphate Buffered Saline (PBS)	(Invitrogen, catalog number: 10010023)
Skeletal muscle dissociation kit	(Miltenyi Biotec, catalog number: 130-098-305)
Thrombin	(Stago, catalog number: HT1002a)
Tranexamic acid	(Sigma-Aldrich, catalog number: 857653)
Triton X-100 (10% in water)	(Sigma-Aldrich, catalog number: 93443)
Trypan Blue	(Fisher Scientific, catalog number: 15-250-061)
Trypsin/EDTA	(Invitrogen, catalog number: 15090046)
Tween-20	(Acros, catalog number: 233360010)
Ultrapure water with 0.1% gelatin	(Millipore, EmbryoMax, catalog number: ES 006B)
Ultroser G serum substitute	(Sartorius, catalog number: 15950-017)
**Antibodies**	
Alexa Fluor 488 goat anti-mouse	(Invitrogen, catalog number: A-11029)
Alexa Fluor 633 goat anti-rabbit	(Invitrogen, catalog number: A21070)
Anti-(human) CD31/PECAM-1 antibody	(Santa Cruz, catalog number: sc-376764)
Anti-(human) desmin antibody	(Invitrogen, catalog number: D1033)
Anti-(human) phalloidin-iFluor 488	(Abcam, catalog number: ab176753)
Anti-(human) tropomyosin antibody	(Invitrogen, catalog number: T9283)
Anti-(human) vWF antibody	(Dako, catalog number: A0082)
APC anti-human CD31 antibody	(Thermo Fisher, catalog number: 17-0319-42)
APC anti-human CD56 antibody	(Thermo Fisher, catalog number: A15704)

### 2.1 Buffer and media compositions

#### 2.1.1 Ammonium-Chloride-Potassium lysing buffer

- 150 mM NH_4_Cl
- 10 mM KHCO_3_
- 0.1 mM EDTA
→ Filter sterilize (0.22 µm filter).

#### 2.1.2 Blocking buffer

PBS supplemented with:

- 1% (w/v%) BSA

- 0.2% Triton X-100

#### 2.1.3 Cell sorting buffer

- 90% PBS
- 10% FBS
→ Filter sterilize (0.22 µm filter).

#### 2.1.4 Skeletal muscle growth medium

DMEM Glutamax supplemented with:

- 10% FBS

- 1% Ultroser G serum substitute

- 0.1% gentamicin

→ Filter sterilize (0.22 µm filter).

#### 2.1.5 Skeletal muscle fusion medium

DMEM Glutamax supplemented with:

- 10 ng/ml hEGF

- 10 μg/ml insulin

- 50 μg/ml BSA

- 50 μg/ml gentamicin

→ Filter sterilize (0.22 µm filter).

## 3 Harvest of skeletal muscle tissue

1. Disinfect the area of dissection with 70% ethanol.
2. Make a small longitudinal incision using sterile forceps.
3. Dissect at least 1 g of skeletal muscle tissue (recommended: vastus lateralis muscle).
4. Sterilize the muscle tissue by holding it for 3 s in 70% ethanol.
5. Wash the tissue 3 times for 5 min with PBS.
6. Place the tissue in DMEM+1% P/S until tissue digestion.

## 4 Automated enzymatic and mechanical tissue digestion using gentleMACS™ Octo Dissociator with Heaters

1. Cut the tissue into small pieces (2–4 mm) using sterile scissors and scalpel blades.
2. Prepare the enzyme mix from Miltenyi Biotec skeletal muscle dissociation kit.

Preparation for 1 g muscle tissue:

• 200 μl of Enzyme D
• 50 μl of Enzyme P
• 36 μl of Enzyme A

Mix all three enzymes in 4.70 ml of DMEM (pre-warmed at 37°C).

3. Transfer muscle pieces with a sterile spatula into a gentleMACS™ C Tube containing the enzyme mix and close tube tightly.
4. Place the C Tube upside down onto the gentleMACS™ device and add the heater.
5. Run the program 37C_mr_SMDK_1 on the gentleMACS™ Octo Dissociator with Heaters.

## 5 Seeding of isolated (unsorted) cell population

1. Coat a T75 cell culture flask by incubating with 6 ml of Ultrapure water with 0.1% gelatin for 30 min at room temperature (RT).
2. Remove the coating solution from the cell culture flask.
3. Add 14 ml of EGM-MV medium and store the flask in the incubator (37°C, 5% CO_2_).
4. Perform a short centrifugation step of the gentleMACS™ C Tube to collect the single cell suspension at the tube bottom.
5. Resuspend the cells and apply the suspension to a 70 µm cell strainer.
  Note: Moisturize the cell strainer upfront with 1–2 ml of DMEM.
6. Wash the cell strainer with 10 ml of DMEM.
7. Discard the cell strainer and centrifuge the cell suspension for 20 min at 300 × g.
8. Aspirate the supernatant carefully.
9. Resuspend the cell pellet in 1 ml DMEM+10% FBS.
10. Apply the cell suspension to a 40 μm cell strainer.
  Note: Moisturize the cell strainer upfront with 1–2 ml of DMEM.
11. Wash the cell strainer with 10 ml of DMEM.
12. Centrifuge the cell suspension for 10 min at 300 × g.
13. Resuspend the cell pellet in 1 ml Ammonium-Chloride-Potassium (ACK) lysing buffer (see Section 2.1) for 30 s at RT to lyse erythrocytes.
14. Inactivate the reaction by adding 9 ml DMEM+10% FBS.
15. Perform a centrifugation step for 10 min at 300 × g.
16. Discard the supernatant and resuspend the cell pellet in 1 ml EGM-MV medium.
17. Seed the cells in 15 ml EGM-MV medium in the coated T75 culture flask.
18. Refresh the EGM-MV medium every second day until cells reach approximately 80% confluency.

## 6 Magnetic-activated cell sorting of isolated cells

At a cell confluency of approximately 80%, detach the cells with 5 ml 0.125% Trypsin-EDTA for 3 min at 37°C, followed by inactivation with 15 ml DMEM+10% FBS. Centrifuge the cell suspension for 10 min at 300 × g, resuspend the cells in EGM-MV medium and determine the cell number using an automated cell counter.

Note: Alternatively, determine the cell number using a Burker cell counting chamber.

### 6.1 Depletion of CD45^+^ cells

(Adapted from Miltenyi Biotec protocol)

1. Resuspend the cell pellet (up to 10^7^ cells) in 80 µl cell sorting buffer (see Section 2.1).
2. Add 20 µl of anti-human CD45 MicroBeads and incubate for 15 min at 4°C.
3. Add 1–2 ml of cell sorting buffer to wash the cells.
4. Centrifuge the suspension for 10 min at 300 × g.
5. Discard the supernatant and resuspend the cell pellet in 500 µl cell sorting buffer.
6. Place an LS magnetic column onto a MACS™ separator using a magnet of respective size.
7. Moisturize the magnetic column with 3 ml of cell sorting buffer.
8. Apply 500 µl of the labeled cell suspension onto the column and let it completely run through the column.
9. Wash the magnetic column three times with 3 ml cell sorting buffer.
10. Centrifuge the collected flow-through for 10 min at 300 × g and continue with Section 6.2.
  Note: The column with CD45^+^ cells can be discarded.

### 6.2 Enrichment of CD31^+^ cells (microvascular endothelial cells)

(Adapted from Miltenyi Biotec protocol)

1. Coat a T25 culture flask by adding 2 ml Ultrapure water with 0.1% gelatin for 30 min at RT.
2. Remove the coating solution from the cell culture flask.
3. Add 5 ml of EGM-MV medium and store the flask in the incubator (37°C, 5% CO_2_).
4. Resuspend the CD45^−^ cell pellet (obtained in Section 6.1) (up to 10^7^ cells) in 80 µl cell sorting buffer.
5. Add 20 µl of anti-human CD31 MicroBeads and incubate for 15 min at 4°C.
6. Add 1–2 ml of cell sorting buffer to wash the cells.
7. Centrifuge the cell suspension for 10 min at 300 × g.
8. Discard the supernatant and resuspend the cell pellet in 500 µl cell sorting buffer.
9. Place an MS magnetic column onto a MACS™ separator using a magnet of respective size.
10. Moisturize the magnetic column with 500 µl cell sorting buffer.
11. Apply 500 µl of the labeled cell suspension onto the column and let it completely run through the column.
12. Wash the column three times with 500 µl cell sorting buffer.
13. Centrifuge the collected flow-through for 10 min at 300 × g and keep on ice until continuing with Section 6.3.
  Note: This cell fraction represents the CD31^−^ cells, which will be used for the subsequent sorting step for CD56 (see Section 6.3).
14. Place the MS column containing accumulating CD31^+^ cells onto a 15 ml-falcon tube.
15. Apply 1 ml cell sorting buffer onto the column and collect magnetically labeled CD31^+^ cells by rapidly pushing the plunger into the column.
16. Centrifuge the CD31^+^ cell suspension for 10 min at 300 × g.
17. Resuspend the cell pellet in 1 ml EGM-MV medium and seed the cells in the coated T25 culture flask.
  Note: To increase the yield of CD31^+^ cells, we recommend repeating Section 6.2 as a purification step of isolated microvascular ECs once cells have reached approximately 80% confluency. Post a second MAC sorting, culture cells until approximately 80% confluency and subsequently proceed with Section 7.

### 6.3 Enrichment of CD56^+^ cells (myoblasts)

(Adapted from Miltenyi Biotec protocol)

1. Coat a T175 culture flask by adding 15 ml Ultrapure water with 0.1% gelatin for 30 min at RT.
2. Remove the coating solution from the cell culture flask.
3. Add 30 ml of Skeletal Muscle Growth Medium (SkGM, see Section 2.1) and store the flask in the incubator (37°C, 5% CO_2_).
4. Resuspend the cell pellet (up to 10^7^ cells) from Section 6.2 in 80 µl cell sorting buffer.
5. Add 20 µl of anti-human CD56 MicroBeads and incubate for 15 min at 4°C.
6. Add 1–2 ml of cell sorting buffer to wash the cells.
7. Centrifuge the suspension for 10 min at 300 × g.
8. Discard the supernatant and resuspend the cell pellet in 500 µl cell sorting buffer.
9. Place an LS magnetic column onto a MACS™ separator using a magnet of respective size.
10. Moisturize the magnetic column with 3 ml cell sorting buffer.
11. Apply 500 µl of the labeled cell suspension onto the column and let it completely run through the column.
12. Wash the column three times with 3 ml cell sorting buffer.
13. Store the collected flow-through on ice until CD56^+^ cells are collected.
  Note: This cell fraction represents the CD56^−^ cells.
14. Place the LS column containing accumulating CD56^+^ cells onto a 15 ml-falcon tube.
15. Apply 5 ml of cell sorting buffer onto the column and collect magnetically labeled CD56^+^ cells by rapidly pushing the plunger into the column.
16. Centrifuge both cell suspensions (CD56^+^ and CD56^−^ separately) for 10 min at 300 × g.
17. Resuspend each cell pellet in 1 ml SkGM and seed the CD56^+^ cells in the coated T175 culture flask.
  Note: For CD56^−^ cells, no coating is required.
18. Refresh the culture medium every second day until cells reach approximately 80% confluency.
19. At a cell confluency of approximately 80%, harvest the cells and proceed with the characterization of the cells (Section 7).

## 7 Characterization of isolated endothelial cells and myoblasts

### 7.1 Immunofluorescence staining (qualitative)

For a qualitative characterization of isolated cells, perform an immunofluorescence staining. The following protocol describes the characterization of isolated ECs using the endothelial cell markers CD31 and von Willebrand factor (vWF). Isolated myoblasts are characterized by the expression of desmin.

1.   Seed 5,000 cells per well in a 24-well plate.
2.   Culture cells until:
  (i) ECs: 90%–100% confluency.
    (ii) myoblasts: 50%–60% confluency.
3.   Rinse the cells 3 times for 5 min with PBS and fix:
  (i) ECs: for 10 min with 4% paraformaldehyde at RT.
    (ii) myoblasts: for 10 min with a 1:1 solution of acetone: methanol at −20°C.
4.   Wash the cells 3 times for 5 min with PBS.
5.   Incubate the cells for 1 h with blocking buffer (see Section 2.1) at RT.
6.   Incubate overnight at 4°C with the respective primary antibody:
  (i) ECs: mouse anti-human CD31, rabbit anti-vWF (10 µg/ml in blocking buffer).
    (ii) myoblasts: mouse anti-human desmin, (1:100 in blocking buffer).
      Note: Keep one well on blocking buffer as a negative control.
7.   Wash the cells 3 times for 5 min with PBS.
8.   Incubate the cells for 30 min at RT with the respective secondary antibody:
  (i) ECs: Alexa Fluor 488-labeled goat anti-mouse antibody, Alexa Fluor 488-labeled goat anti-rabbit antibody (1:200 in PBS).
    (ii) myoblasts: Alexa Fluor 488-labeled goat anti-mouse antibody (1:200 in PBS).
9.   Wash the cells 3 times for 5 min with PBS.
10. Incubate the cells for 30 min at RT with DAPI solution (1:10,000 in PBS).
11. Replace the DAPI solution with PBS.
12. Image the cells using a fluorescence microscope.

### 7.2 Flow cytometry analysis (quantitative)

**FIGURE 3:**
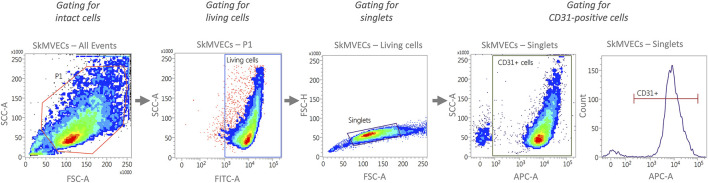
Representative plots generated by flow cytometry showing the recommended gating strategy for the characterization of isolated SkMVECs. First, intact cells are selected by plotting forward scatter area (FSC-A) versus side scatter area (SSC-A), with P1 representing the cell population excluding debris. Next, the cells are further gated for living cells by using FITC-channel for detecting Calcein AM labeled cells. Of the living cells, singlets are selected by plotting forward scatter height (FSC-H) versus forward scatter area (FSC-A). Finally, the singlets are further gated for APC-labeled CD31-positive cells using the APC channel.

For quantitative analysis of isolated cells, perform flow cytometry analysis. Isolated SkMVECs can be assessed according to the percentage of CD31^+^ cells and isolated myoblasts according to the expression of CD56. The preparation for flow cytometry, including a recommended gating strategy (Figure 3), is described in the following.

1. Centrifuge cells (approximately 2.0 × 10^5^ cells per tube) for 5 min at 300 × g in three falcon tubes to prepare:
  - cells stained for the respective cell marker (here: CD31, CD56).
  - fluorescence-minus-one (FMO) control.
  - negative control of Calcein AM staining.
    Note: Calcein AM staining is applied for the gating for living cells.
2. Preparation of stained cells and FMO control for APC:

Resuspend each cell pellet in 100 µl cold PBS+10% FBS. Add 5 µl of APC-labeled anti-human CD56 (for myoblasts) or CD31 (for SkMVECs) antibody to the cells to be stained. No antibody is added to the FMO control tube. Incubate for 30 min at 4°C.

3. Preparation of negative control for Calcein AM staining:

Resuspend the cell pellet in 1 ml PBS, transfer it to an Eppendorf tube, and incubate the cell suspension for 10 min at 65°C.

4. Wash all three samples by adding 1 ml of cold PBS+10% FBS and centrifuge for 10 min at 300 × g.
5. Resuspend each cell pellet in 1 ml of Calcein AM staining solution (0.1 µM in PBS).
6. Incubate for 15–20 min at RT in the dark.
7. Centrifuge the cells for 10 min at 300 × g.
8. Resuspend each cell pellet in 200 µl of cold PBS+3% FBS and transfer to a FACS tube.
9. Analyze cells with a flow cytometer using the recommended gating strategy as shown in Figure 3.

## 8 Functional characterization of isolated cells

### 8.1 Functional characterization of isolated endothelial cells

#### 8.1.1 Endothelial network formation on Matrigel (tube formation assay)

1. Thaw the required amount of growth factor reduced Matrigel in the fridge. Calculate for each well of a 48-well plate 150 μl. Once the Matrigel is liquid, store it on ice.
2. Place the 48-well plate and pipette tips in the fridge before the assay.
3. Add 150 μl growth factor reduced Matrigel per well and place for 30 min in the incubator (37°C, 5% CO_2_).
4. Detach cells using 0.125% trypsin/EDTA and incubate for 3 min in the incubator (37°C, 5% CO_2_).
5. Inactivate trypsin with DMEM+10% FBS.
6. Centrifuge for 5 min at 200 × g and resuspend the cell pellet in EGM-MV medium.
7. Determine the cell number and pellet down the required number of cells: 45,000 cells per well of a 48-well plate.
8. Resuspend the cells in 250 μl EGM-MV medium per well of a 48-well plate.
9. Seed the cells on solidified Matrigel and place them in the incubator (37°C, 5% CO_2_).
 Note: Tubes will start forming within 2 h post-seeding and can be visualized up to 12 h post-seeding. Cells should not be kept longer in culture than 24 h.
10. For fixation of the tubes, wash for 5 min with PBS and incubate with 4% paraformaldehyde for 10 min at RT. Wash 3 times with PBS.

#### 8.1.2 Endothelial sprouting formation in fibrin hydrogel (spheroid-based sprouting assay)

1. Detach cells using 0.125% trypsin/EDTA and incubate for 3 min in the incubator (37°C, 5% CO_2_).
2. Determine the cell number and pellet down the required number of cells.
3. Resuspend 100,000 ECs in EGM-MV medium containing 20% methylcellulose.
4. Transfer to a multichannel pipette reservoir and pipet spheroids of 25 µl each on non-adherent plastic dishes to generate spheroids containing each 1,000 cells.
5. Turn the plate upside down and culture at 37°C and 5% CO_2_ overnight.
6. Harvest the spheroids by gently washing the plastic dishes with PBS containing 10% FBS.
7. Transfer to a 50 ml tube and centrifuge first 5 min at 300 × g followed by 3 min at 500 × g at RT.
8. Aspirate the supernatants carefully.
9. Resuspend the spheroids gently in 1 ml of 2 mg/ml fibrinogen solution.
10. Add 4 U/ml thrombin and pipet 2 times up and down to mix.
11. Transfer into a 12-well plate rapidly.
12. Place the 12-well plate for 1 h at 37°C to allow the fibrin (1 mg/ml) hydrogel to solidify.
13. Add 1 ml EGM-MV medium containing tranexamic acid (final concentration 400 μM) and aprotinin (final concentration 92.5 μg/ml).
14. After 24 h, fix the spheroids for 30 min at RT with 4% paraformaldehyde.
15. Wash 2 times with PBS.
16. Permeabilize with PBS containing 1% Tween20 for 10 min.
17. Stain for 1 h at RT with phalloidin (1:250 in PBS) and DAPI (1:10,000 in PBS).
18. Wash 3 times with PBS.
19. Image using fluorescence microscopy.

### 8.2 Functional characterization of isolated myoblasts

#### 8.2.1 Myotube formation in a 24-well plate (fusion assay)

(Adapted from protocol described in Gholobova et al. (2019)).

1. Seed 50,000 cells per well in a 24-well plate.
2. Continue to expand the cells, refresh SkGM every 2 days until cells reach 90%–100% confluency.
3. Change the medium to Skeletal Muscle Fusion medium (SkFM, see Section 2.1) for 4 days.
4. Refresh the medium every second day.
5. After 7 days, fix the cells with 4% paraformaldehyde for 10 min at RT.
6. Rinse the cells 3 times for 5 min with PBS.
7. Fix the cells with ice-cold methanol for 10 min at −20°C.
8. Rinse the cells 3 times for 5 min with PBS.
9. Incubate the cells for 1 h with blocking buffer at RT.
10. Incubate overnight at 4°C with a primary mouse anti-human sarcomeric tropomyosin antibody (1:100 in blocking buffer).
11. Rinse the cells 3 times for 5 min with PBS.
12. Incubate with Alexa Fluor 488-labeled goat anti-mouse antibody (1:200 in blocking buffer) for 1 h at RT.
13. Rinse the cells 3 times for 5 min with PBS.
14. Incubate for 30 min at RT with DAPI solution (1:10,000 in PBS).
15. Image DAPI-stained nuclei and tropomyosin-positive myotubes using fluorescence microscopy.

## 9 Lentiviral vector transduction of isolated skeletal muscle microvascular endothelial cells

1. Seed SkMVECs in a T25 (approximately 200,000 cells) and let cells proliferate until 80% cell confluency. Refresh EGM-MV medium every second day.
2. Remove the medium and add 50 μl of lentiviral vector (5.2 × 108 pg p24/ml) in 2 ml of EGM-MV medium to the cells.

Viral vector: pCH-EF1a-eGFP-Ires-Puro Note: Retroviral vectors were produced by the Leuven Viral Vector Core as described in Ibrahimi et al. (2009). The transfer plasmid used encoded eGFP IRES Puromycin resistance cassette driven by the human elongation factor 1 alfa promoter (EF1a) (pCH-EF1a-eGFP-Ires-Puro). The integrity of all plasmids was verified by digestion and DNA sequencing prior to vector production. Titer (measured by quantifying p24 enzyme linked immunosorbent assay (ELISA) (HIV-1 core profile ELISA, DuPont)) and transducing units following transduction of 293T cells: 5.2 × 10 8 pg p24/ml–2.1 × 10 9 TU/ml.

3. Allow viral vector to transduce cells for 24 h at 37°C, 5% CO_2_.
4. Remove EGM-MV medium supplemented with viral vector and perform a selection of the transduced cells by incubating cells for 48 h in EGM-MV medium supplemented with 2 µg/ml puromycin.
5. Use selected cells for subsequent experiments or cryo-preserve in liquid nitrogen.

## 10 Alternatives

If for the application of this protocol specific products or devices of Miltenyi Biotec are not available, following alternatives may be applied:

### 10.1 Magnetic-activated cell sorting buffer

As an alternative to using MACS buffer, PBS+10% FBS can be used for the cell sorting steps. In addition, for the final sorting steps before seeding the cells in culture plates, the respective medium for the cell type of interest can be used for this sorting step.

### 10.2 Magnetic-activated cell sorting enzymes

As an alternative for the enzymes A, D and P which come along with the skeletal muscle dissociation kit of Miltenyi Biotec, a combination of the two enzymes Collagenase II (1.5 mg/ml) and Dispase II (4 mg/ml) can be used for the tissue dissociation step using gentleMACS™ Octo Dissociator with Heaters.

### 10.3 GentleMACS™ Dissociator

If the gentleMACS™ Dissociator (with or without heaters) is not available, we advise to use the manual dissociation procedure described in Müntefering et al. (2019) as an alternative. Briefly, the tissue pieces are resuspended in the dissociation enzyme solution and placed in the incubator (37°C, 5% CO_2_) for 1.5 h. Every 20 min, the suspension is mixed using a 1 ml syringe.

## 11 Representative results

**FIGURE 4:**
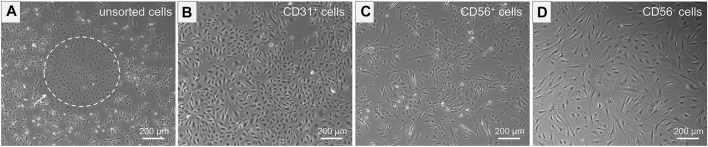
Morphology of isolated cells. Microscopic images represent the morphology of the different isolated cell types. **(A)** Unsorted cells containing multiple cell types of distinct morphologies. Dashed white circle indicates SkMVECs. **(B)** Isolated CD31^+^ cells, SkMVECs, displaying a typical round, cobblestone-shaped morphology. **(C)** Isolated CD56^+^ cells, satellite cell-derived myoblasts, appearing as more elongated, oval-shaped cells. **(D)** Remaining CD56^−^ cells, including fibroblasts and pericytes, displaying a larger cell size and irregular shape, partially with protrusions.

The unsorted cell population, obtained after tissue digestion, was cultured in a cell culture flask until a cell confluency of approximately 80% was reached. The distinct morphologies of isolated cells prior to MAC sorting observed in microscopic images of unsorted cells is shown in Figure 4A. SkMVECs were found to grow in clusters between the surrounding cell types (marked with a white dashed circle, Figure 4A). Post MAC sorting, the separated cell types, SkMVECs, myoblasts, and the remaining CD56^−^ cell fraction (primarily fibroblasts and pericytes) were subsequently cultured in different cell culture flasks in their respective culture medium. The label of the cells indicates the marker that was used for MAC sorting: microvascular ECs are indicated as CD31-positive (CD31^+^), myoblasts as CD56-positive (CD56^+^), and the remaining CD31-negative and CD56-negative (short: CD56^−^) cell fraction containing cells such as fibroblasts and pericytes. Isolated SkMVECs can be recognized by their cobblestone shape as demonstrated in Figure 4B. Isolated satellite cell-derived myoblasts display a typical spindle-shaped morphology (Figure 4C). Lastly, the remaining CD56^−^ cells appear as larger, irregularly shaped cells, partially displaying protrusions (Figure 4D).

**FIGURE 5:**
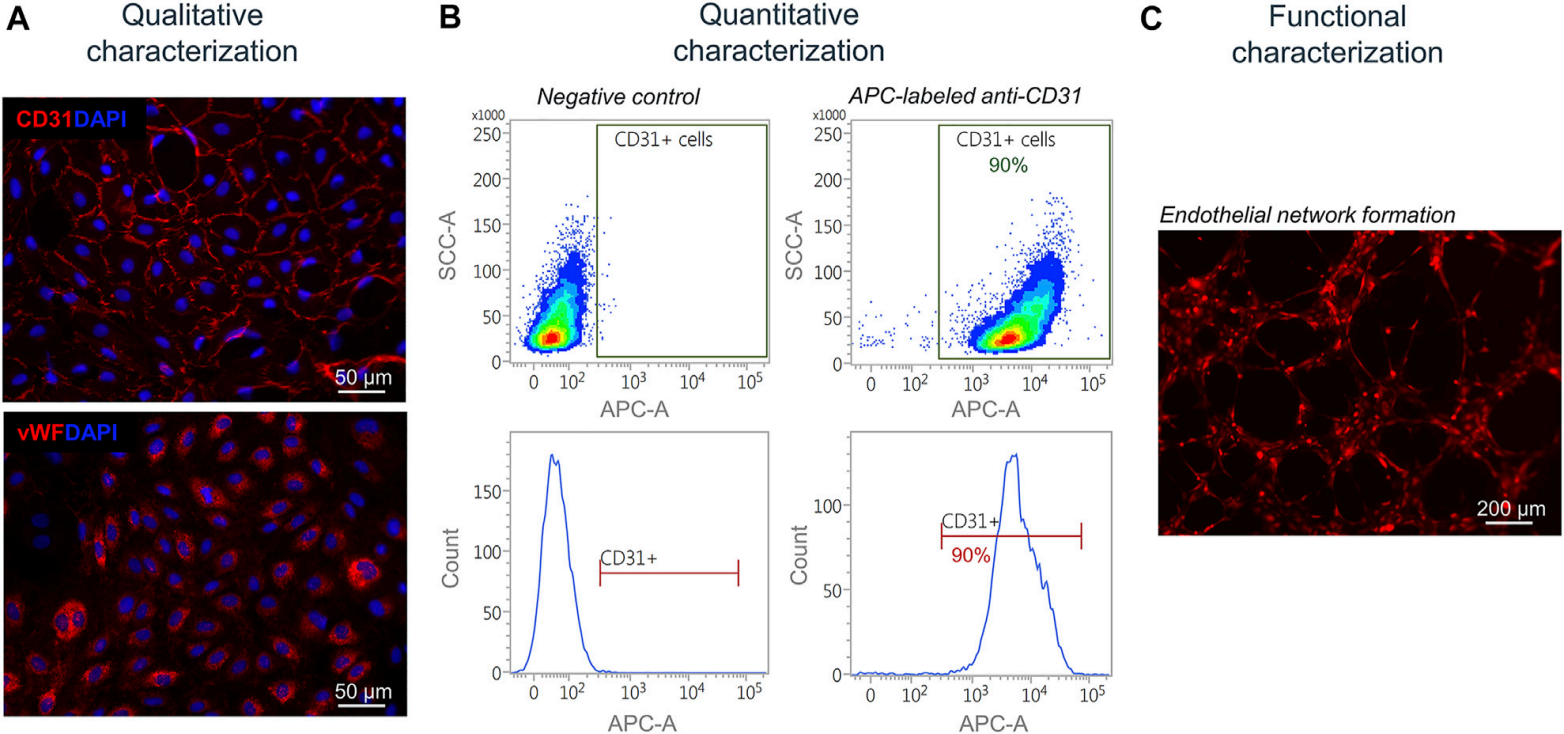
Characterization of isolated SkMVECs. **(A)** Immunofluorescence staining of isolated SkMVECs for the endothelial cell markers CD31 (pseudo-color red) and vWF (pseudo-color red). Nuclei are DAPI-stained (blue) **(B)** Quantitative characterization by flow cytometry for CD31. **(C)** Tube formation assay of GFP-labeled isolated SkMVECs on growth factor reduced Matrigel imaged after 4 h showing endothelial networks formed *in vitro* (pseudo-color red).

Next, isolated and separated cells were characterized qualitatively, quantitatively, and functionally. Isolated SkMVECs were fixed and qualitatively characterized by immunofluorescence staining for the two endothelial cell markers CD31 and vWF (Figure 5A). For a quantitative characterization, the number of CD31^+^ cells was determined by flow cytometry using an APC-labeled antibody against human CD31. Results showed a purity of about 90% of isolated SkMVECs after a second MAC sorting (Figure 5B). By performing a tube formation assay, it was demonstrated that isolated SkMVECs form endothelial networks *in vitro* (Figure 5C).

**FIGURE 6:**
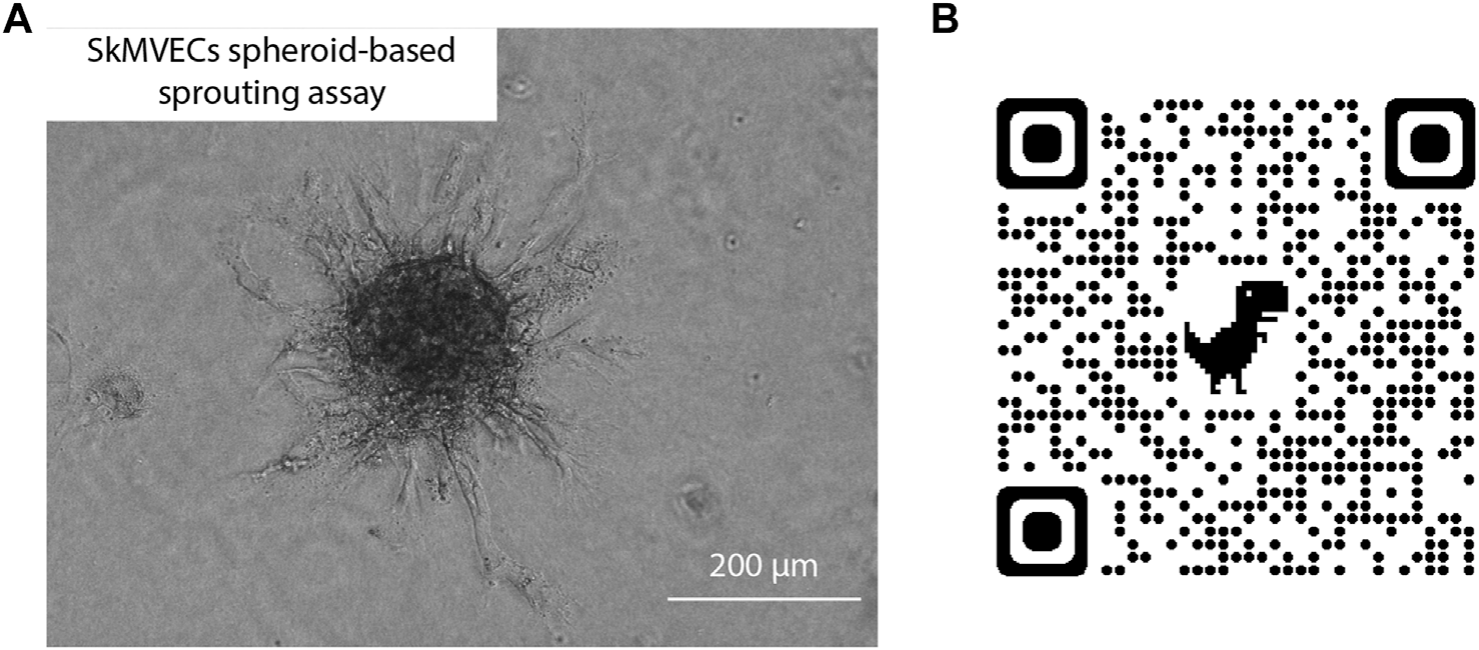
**(A)** Light microscopic image of human SkMVECs spheroid-based sprouting assay in a fibrin hydrogel analyzed after 24 h. **(B)** QR-code linked to 3D visualization of spheroid-based sprouting assay captured with confocal microscopy.

In addition, a spheroid-based sprouting assay was performed. This assay is a widely used *in vitro* angiogenesis assay that allows the evaluation of the sprout formation capacity of the isolated endothelial cells. Similar to the tube formation assay, this sprouting assay can be used to quantify and evaluate angiogenesis *in vitro*. The advantage of the sprouting assay over the tube formation assay is the 3D culture technique which better reflects the *in vivo* angiogenesis. Using this assay, the sprouting of the isolated SkMVECs was shown (Figure 6).

**FIGURE 7:**
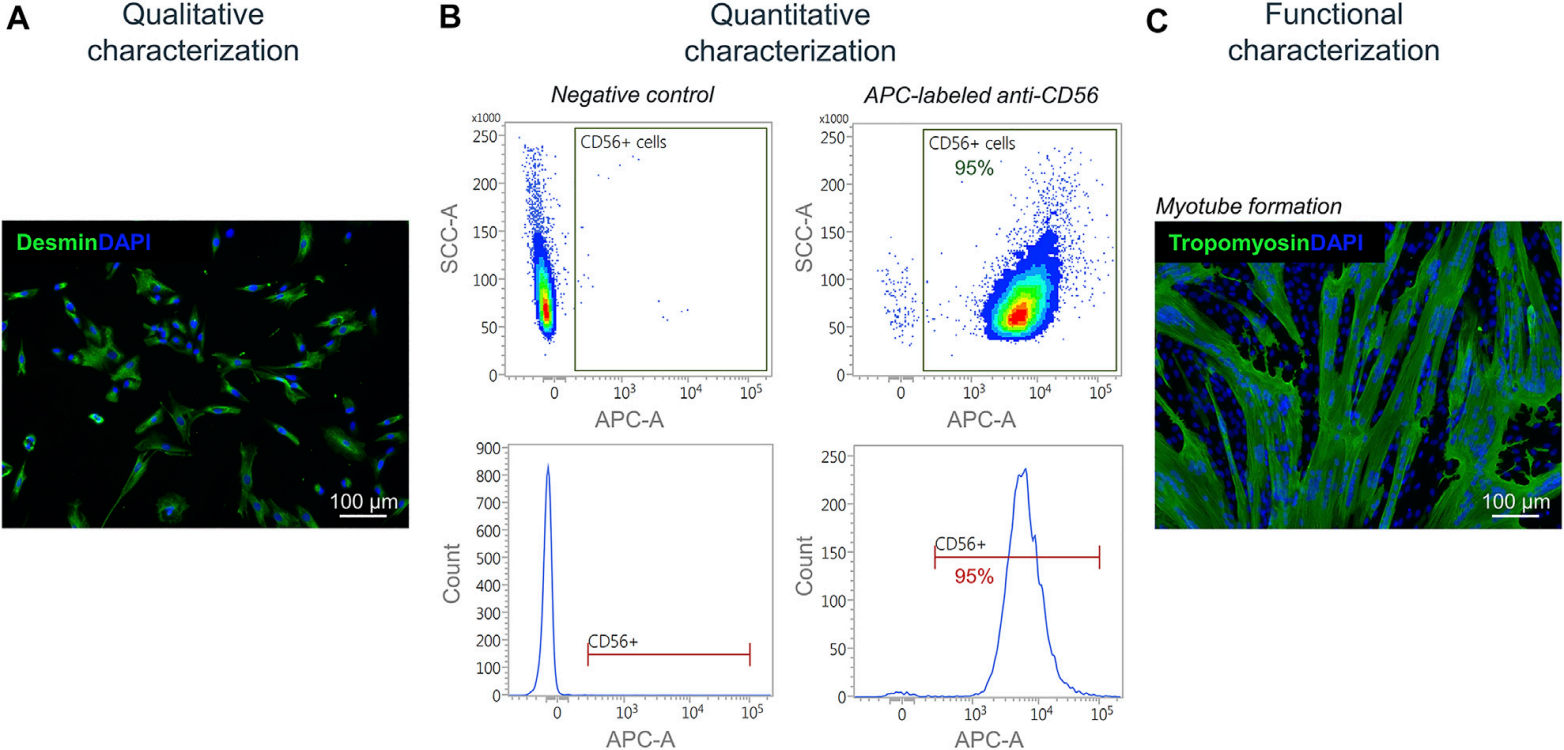
Characterization of isolated myoblasts. **(A)** Immunofluorescence staining of isolated satellite cell-derived myoblasts for the muscle cell marker desmin (green). Nuclei are DAPI-stained (blue) **(B)** Quantitative characterization by flow cytometry for CD56. **(C)** Fusion assay of isolated satellite cell-derived myoblasts showing the formation of multinucleated (DAPI-stained, blue) tropomyosin-positive myotubes (green) after 7 days of culturing *in vitro*.

Isolated satellite cell-derived myoblasts were qualitatively characterized by immunofluorescence staining for the muscle-specific marker desmin (Figure 7A). For a quantitative characterization, flow cytometry was performed using an APC-labeled antibody against human CD56. Results showed a purity of cells of up to 95% post MAC sorting (Figure 7B). By performing a fusion assay, it was demonstrated that the isolated satellite cell-derived myoblasts fuse and form multinucleated myotubes *in vitro* (Figure 7C).

**FIGURE 8:**
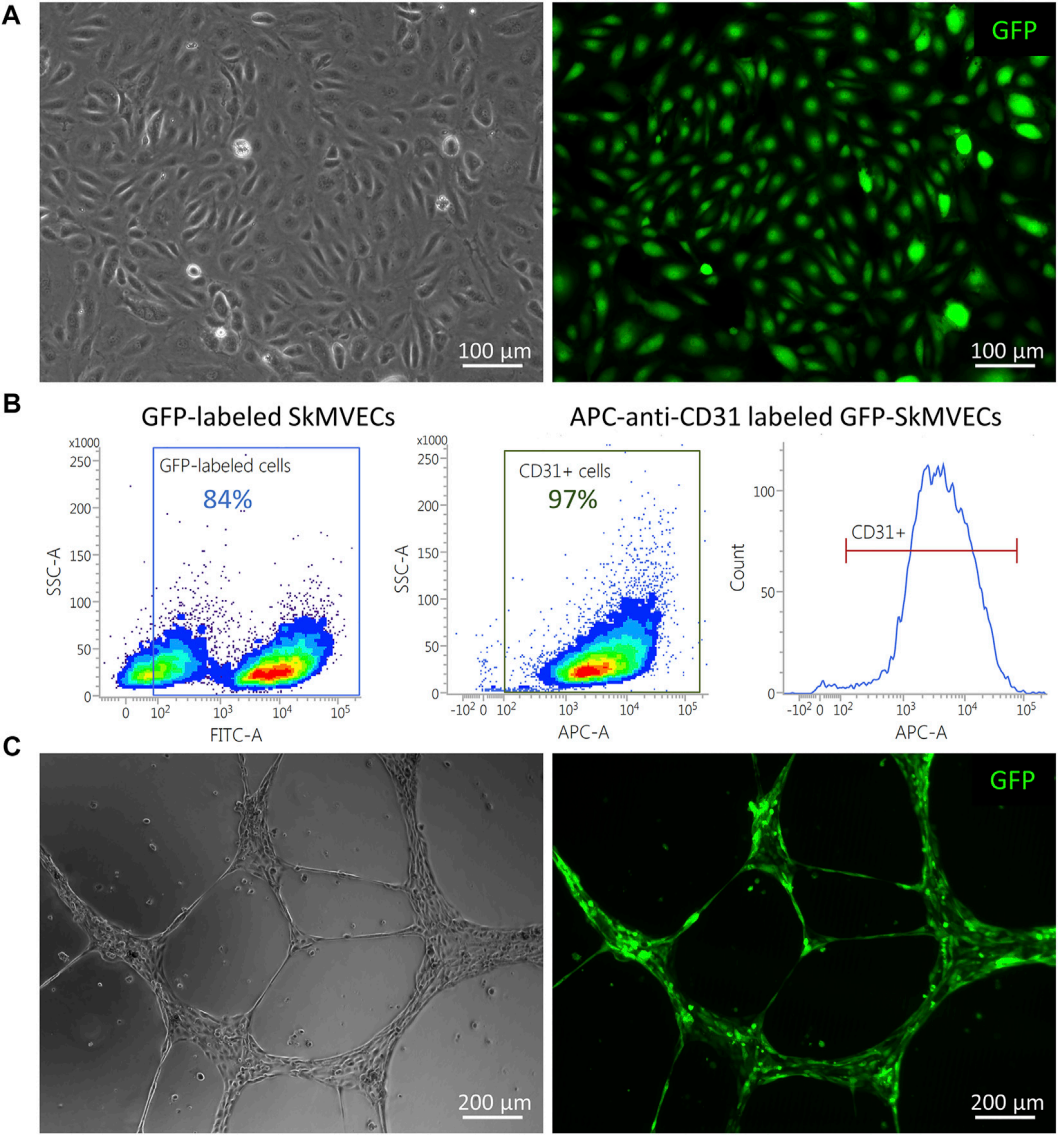
GFP-expressing SkMVECs. **(A)** Microscopic image of cobblestone-shaped isolated SkMVECs (left image) showing GFP expression post viral vector transduction (green, right image). **(B)** Flow cytometry analysis result showing out of 84% GFP-expressing cells, 97% are positive for the endothelial cell marker CD31. **(C)** Tube formation assay on growth factor reduced Matrigel showing formation of endothelial networks (left image) by isolated GFP-expressing SkMVECs (green, right image).

For the use of isolated SkMVECs to create vascularized 3D bioartificial tissue constructs, it can be advantageous to use fluorescent protein expressing cells, especially for multilineage tissue engineering which requires multi-staining to detect the different cell types. Therefore, we tested whether the isolated SkMVECs can successfully be transduced with a lentiviral vector encoding for green fluorescent protein (GFP). Immunofluorescent imaging demonstrated GFP-expression of transduced SkMVECs (Figure 8A). Flow cytometry analysis confirmed 84% GFP-expressing cells with a high purity of 97% CD31-expressing cells (Figure 8B). In addition, performing a tube formation assay verified that the endothelial network formation capacity of GFP-expressing SkMVECs was not affected by the lentiviral vector transduction (Figure 8C).

## 12 Discussion

The vascularization of tissue-engineered constructs remains a key challenge in the field of skeletal muscle tissue engineering. So far, HUVECs have been the predominantly used EC type for generating vascular networks within an engineered tissue. These cells are the best-characterized EC type and can easily be isolated from human umbilical cords, a non-invasive harvesting method. However, HUVECs are not a representative EC type for every application (Hauser et al., 2017). ECs are known to display a great heterogeneity (Garlanda and Dejana 1997) and show tissue- and organ-specific characteristics (Rafii et al., 2016). Both the expression profile and the functional responses have been reported to vary between ECs from different origins (Hauser et al., 2017). For example, Dib et al. (2012) compared the proteome profile of HUVECs to human microvascular pulmonary and dermal ECs, and reported a noticeable difference, reflecting different biological properties. Therefore, the use of skeletal muscle-specific microvascular endothelial cells (SkMVECs) may have great potential for improving *in vitro* pre-vascularization of engineered muscle. A limitation thus far has been the lack of a reliable protocol to isolate human SkMVECs. Engineering a vascular network within a tissue construct can be achieved by co-culturing ECs with myoblasts in a 3D co-culture setting. The endothelial cells have the capacity to self-assemble into a vascular network, a process called vasculogenesis. Considering the tissue-specificity of ECs, different groups have started investigating the interaction between endothelial cells and muscle satellite cells (Chiristov et al., 2007; Osaki et al., 2018; Verma et al., 2018). For studying this cross-talk and setting up a co-culture, it may be beneficial to obtain both cell types from the same biological origin. For this, we developed a protocol to co-isolate microvascular endothelial cells and myoblasts from human skeletal muscle tissue.

For the isolation of myoblasts from skeletal muscle tissue, several protocols exist for human tissue and tissues of another origin. In contrast, the number of protocols that can be found for the isolation of microvascular endothelial cells is limited. Focusing on microvascular endothelial cells from human tissues, protocols have been published for the isolation of, inter alia, dermal microvascular ECs (Mason et al., 2007), pulmonary microvascular ECs (Hewett and Murray 1993), brain microvascular ECs (Navone et al., 2013), or microvascular ECs from adipose tissue (Hewett et al., 1993). However, no protocol has been described for the isolation of human skeletal muscle-derived microvascular ECs.

With this work, we not only demonstrate the successful isolation of SkMVECs, but also the simultaneous co-isolation of human satellite cell-derived myoblasts. Both isolated cell types were characterized by immunofluorescence staining for specific cell markers. SkMVECs were shown to be positive for CD31, the platelet endothelial cell adhesion molecule (PECAM-1), and for the von Willebrand factor (vWF). Isolated satellite cell-derived myoblasts were positive for desmin. These results were verified and quantified by performing flow cytometry, showing that approximately 95% of myoblasts were positive for CD56 and approximately 90% of isolated ECs positive for CD31 post a second sorting for CD31. This second CD31 MAC sorting step was performed as a purification step to achieve a higher yield of CD31^+^ cells, since after the first sorting only around 70% of the cells were CD31^+^ (data not shown). If the purity of isolated SkMVECs is lower than expected, an additional purification step (third MAC sorting) is recommended to be performed. After the dissociation of muscle tissue, the resulting single cell suspension may also contain leucocytes, erythrocytes, pericytes and fibroblasts, besides the two cell types of interest (SkMVECs and myoblasts) (Birbrair et al., 2013). Therefore, after one or even multiple rounds of MAC sorting, a minor fraction of these other cell types may still be present. In the first MAC sorting step in this protocol, cells of hematopoietic origin are removed by depleting CD45^+^ cells. Since CD45 is not expressed on mature erythrocytes, these cells are removed before the sorting by an incubation step with an ACK lysing buffer. After the removal of these cells and the enrichment of the cells of interest by performing MAC sorting, the remaining CD56^−^ cell fraction primarily represents fibroblasts and/or pericytes. For further application in vascularization research, it may be advantageous to maintain this cell fraction in culture. Indeed, fibroblasts and pericytes have been demonstrated to play important roles in the engineering of microvascular networks *in vitro*, as for example described in Levenberg et al. (2005).

In addition to the qualitative and quantitative characterization, the functional characteristics of the isolated cells, which are of interest for the engineering of vascularized tissue, were evaluated. Isolated SkMVECs were found to self-assemble and form vascular networks when seeded onto growth factor reduced Matrigel. Isolated satellite cell-derived myoblasts were demonstrated to fuse and form multinucleated myotubes. Performing an immunofluorescence staining demonstrated that the formed myotubes were positive for tropomyosin. Although this protocol was developed for human skeletal muscle, it may also be applied for the isolation of SkMVECs and co-isolation of satellite cell-derived myoblasts from skeletal muscle tissue from other species. Furthermore, the described protocol may be applicable to isolate microvascular ECs and surrounding tissue-specific cells of different tissue types by adapting the choice of respective cell-specific markers for MAC sorting.

To conclude, using the developed and in detail described protocol allows the efficient isolation of SkMVECs and satellite cell-derived myoblasts simultaneously from the same human skeletal muscle biopsy. Demonstrated by the functional characteristics, these cells will be of great interest for downstream applications in vascularization research, specifically in skeletal muscle tissue engineering, but also in other domains such as fundamental endothelial cell research.

## Data Availability

The raw data supporting the conclusions of this article will be made available by the authors, without undue reservation.
